# Citrus Alkaline Extract Delayed the Progression of Pulmonary Fibrosis by Inhibiting p38/NF-κB Signaling Pathway-Induced Cell Apoptosis

**DOI:** 10.1155/2019/1528586

**Published:** 2019-02-04

**Authors:** Qi Wu, Yao Zhou, Xian-mei Zhou

**Affiliations:** ^1^Physiology Department, Xuzhou Medical University, Xuzhou 221009, China; ^2^Pathophysiology Department, Xuzhou Medical University, Xuzhou 221009, China; ^3^Department of Respiratory Medicine, Jiangsu Province Hospital of Traditional Chinese Medicine, Nanjing 210029, China

## Abstract

**Objective:**

To investigate the intervention effect and functioning mechanism of citrus alkaline extract (CAE) on bleomycin- (BLM-) induced pulmonary fibrosis in mice.

**Methods:**

42 C57BL/6 male mice were assigned randomly to the normal group, model group, low (16mg/kg), medial (32mg/kg) and high (64mg/kg) CAE dosage groups, prednisone group (6mg/kg), and pirfenidone group (100mg/kg), respectively. One day after model construction, intragastric administration was applied to the mice once a day for 28 days and then killed. Body weights of mice were recorded. Their pulmonary tissues were subjected to HE staining and Masson's staining and then their degree of pulmonary alveolitis as well as pulmonary fibrosis was scored. Contents of hydroxyproline (HYP) and prostaglandin E2 (PGE2) in pulmonary tissues and levels of interleukin-17 (IL-17) in serum and bronchoalveolar lavage fluid (BALF) were determined by ELISA method. Expression of collagen I, collagen III, and Prosurfactant protein C (Pro-SPC) proteins in pulmonary tissue were measured immunohistochemically and that of nuclear transcription factor *κ*B (NF-*κ*B) and vimentin was determined by the immunofluorescence method. Apoptosis of pulmonary tissue was tested by the Tunel staining method, while the expression of MAPK-related protein was recorded by Western Blot assay.

**Results:**

After CAE treatment, the body weight, PGE2 level, and Pro-SPC protein expression of pulmonary fibrosis mice were increased, while the score of pulmonary alveolitis and pulmonary fibrosis, levels of HYP and cell apoptosis, IL-17 contents of serum and BALF in pulmonary tissues, and expression of collagen I, collagen III, vimentin, NF-*κ*B, and p-p38 were reduced.

**Conclusion:**

CAE effectively delayed the progression of BLM-induced pulmonary fibrosis in pulmonary fibrosis mice and a possible mechanism is the inhibition of cell apoptosis of NF-*κ*B/p38-mediated signaling pathway.

## 1. Introduction

Pulmonary fibrosis is a progressive disease that causes death [1]. In recent years, the incidence of pulmonary fibrosis keeps rising with poor prognosis and high mortality [2]. It is currently believed that the main pathological traits of pulmonary fibrosis are characterized by the damage and repair of pulmonary tissues, massive proliferation of pulmonary fibroblasts, and mass secretion and precipitation of collagen matrix [3]. Pulmonary fibrosis progression initiates from the damage of alveolar epithelial cells in response to various stimuli. The damaged alveolar epithelial cells then induced and secreted a variety of cell factors, including transforming growth factors *β*1 (TGF-*β*1) and IL-17 [4]. These cell factors can induce the formation of pulmonary fibroblasts and myofibroblasts focus by stimulating the proliferation of mesenchymal cells, attracting and recruiting pulmonary fibroblasts, and stimulating the epithelial-mesenchymal transition (EMT) of alveolar epithelial cells. Pulmonary fibroblasts and myofibroblasts can secrete a mass of collagen matrix, which consequently leads to the deformation of pulmonary tissues and irreversible damage of pulmonary function, respiratory failure, and eventually death [5].

However, no drug with definite curative effect is available as yet except for lung transplantation. Pulmonary fibrosis is previously cured with glucocorticoids, cyclosporin A, colchicines, and cyclophosphamides. These drugs are denied due to poor curative effect and high side effect [6]. The long-term validity and safety of the new drugs pirfenidone and nintedanib remain ambiguous. Therefore, it is of important significance to screen out drugs with definite curative effects for pulmonary fibrosis treatment [7]. The traditional Chinese medicines are featured by multiple targets and low side effects, which allows us to find more suitable drugs for pulmonary fibrosis treatment.

Citrus peel is the dry mature fruit peel of rutaceous plants and their cultivars, which belong to the Citrus genus and Citrus species. As a frequently-used Chinese medicine, it is widely distributed, abundant and has high pharmaceutical value. Currently it has been confirmed that citrus peel and its bioactive components have anti-inflammatory antioxidative function and now is being widely used for the prevention and treatment of cardiocerebral vascular diseases [8]. However, no study has ever addressed its effect on pulmonary fibrosis as yet. Our research group has conducted numerous studies concerning the effects of Citrus peel and its bioactive components on pulmonary fibrosis treatment. Our previous research showed that the ethanol extract and alkaloids in citrus peel have significant antipulmonary fibrosis activity* in vivo* and* in vitro* [9, 10]. But the functioning mechanism remains to be clarified. In this study, we constructed the pulmonary fibrosis mouse model using the disposable BLM instillation method and employed CAE to treat pulmonary fibrosis interventionally. We observed the effects of CAE on the p38/NF-*κ*B signaling pathway in pulmonary fibrosis mice and determined the functioning mechanism of CAE in pulmonary fibrosis treatment.

## 2. Materials and Methods

### 2.1. Reagents and Instruments

The citrus peel was purchased from Jiangsu Provincial Hospital of Traditional Chinese Medicine. Bleomycin hydrochloride (Nipponkayaku Co., Japan), prednisone tablets (Tianjin Tianyao Pharmacy Co., Ltd, China), and Pirfenidone (BJcontinet Pharmacy Co., Ltd, China) were from commercially available companies. Anti-collagen I antibody, anti-collagen III body, anti-vimentin antibody, anti-Pro-Spc antibody, anti-NF-*κ*B P65 antibody, anti-p-ERK antibody, anti-p-JNK antibody, anti-p-p38 antibody, anti-p38 antibody, and anti-*β*-actin antibody were bought from CST company (America). Mouse IL-17 reagent kit, mouse PGE2 reagent kit, and mouse hydroxyproline reagent kit were purchased from relevant companies in China.

### 2.2. Preparation of CAE

The citrus peel was boiled in ethanol, dissolved in acid, and precipitated in alkali, and then CAE was produced according to a previous paper [9].

### 2.3. Animal Source and Grouping

Healthy male SPF-grade C57BL/6 mice (6-8 weeks old and 18-20g) were used in the experiment. The test animals were supplied by Changzhou CAVENS Experimental Animal Co., Ltd (Changzhou, China). These mice were acclimated at the pediatrics research institute of traditional Chinese medicine, Nanjing University of Chinese Medicine at room temperature and water as well as food was offered ad libitum. The test mice were paralyzed firstly by chloral hydrate via intraperitoneal injection, and then pulmonary fibrosis model mice were constructed by disposable BLM (5mg/kg) instillation. Details about this method can be referred to previous publication [9].

These 42 mice were divided into 7 groups randomly, 6 ones in each group. The normal group was subjected to gastric perfusion by physiological saline. Same procedure was performed to the control group after model construction. For high-dosage group, the mice were subjected to gastric perfusion by 64mg/kg of CAE after model construction. For the medial- and low-dosage groups, mice were subjected to gastric perfusion by 32 mg/kg and 16 mg/kg of CAE after model construction, respectively. The prednisone group was subjected to gastric perfusion by 6mg/kg of prednisone after model construction. The pirfenidone group was subjected to gastric perfusion by 100 mg/kg of pirfenidone after model construction. Gastric perfusion by physiological saline, CAE, prednisone and pirfenidone were applied once a day. The mice were weighed once a week and dosed for 28 days continuously. Fur color, mental state and other general condition of mice were observed daily. At day 28, two hours after dosing the mice were paralyzed and their serum, BALF, and pulmonary tissues were collected and put into -80°C prior to analysis.

### 2.4. Indexes under Investigation and Test Methods

#### 2.4.1. Assessment of Pulmonary Alveolitis and Pulmonary Fibrosis Scores

The pulmonary tissues were fixed, dehydrated, embedded, and then cut into 5 um thick slices. The slices were stained by HE and Masson and their morphologies were observed under an optical microscope. The degree of pulmonary alveolitis and pulmonary fibrosis was assessed according to the methods of Szapiel [11].

#### 2.4.2. Determination of HYP and PGE2 in Pulmonary Tissues

30 mg of pulmonary tissues was homogenized and the supernatant was collected. Absorbance (A) value was measured according to instruction of the ELISA reagent kit. Final contents of HYP and PGE2 were calculated from standard curves.

#### 2.4.3. Determination of IL-17 in Serum and BALF

The test samples were molten at room temperature, incubated, washed, enzymatically catalyzed, color-developed and stopped successively according to the manufacturer's manual on the ELISA reagent kit, and then subjected to absorbance value (A) recording. IL-17 content was calculated according to the standard curve.

#### 2.4.4. Determination of Collagen I, Collagen III, and Pro-SPC Expression by Immune-Histochemical Assay

Embedded mice pulmonary tissues were cut into 4*μ*m thick slices. The resulting slices were dewaxed, and then immersed into 3% H_2_O_2_. The slices were sealed by goat serum and then allowed overnight at 4°C after adding the primary antibody. The second antibody wad added in the second day. The slices were stained by diaminobenzidine, dehydrated, and sealed. Subsequently, they were observed with optical microscope (200×). The yellow brown area was positive area.

#### 2.4.5. Determination of Vimentin and NF-*κ*B Expression by Immunofluorescence Assay

The paraffin section was baked, dewaxed, and then repaired by immersing cells into citrate solution. After being sealed by 10% of goat serum at 37°C for 30 min, the primary antibody was added at a scale of 1:200 and the sections were left overnight at 4°C.In the second day, the sections were washed with PBS and then were incubated for 1h in dark with the second fluorescent antibody. At last the nuclei were stained by DAPI and then sealed by adding fluorescent antiquenching agent. The sections were observed with a fluorescent microscope.

#### 2.4.6. Tunel Staining of Pulmonary Tissues

The paraffin sections were dewaxed and then hydrated. Subsequently, proteinase K working solution and Tunel reaction mixture were added. After drying, 50*μ*l of converter-POD was applied to the specimens. The mixture was covered by cover glass or sealing membrane and then was allowed to react in dark boxes at 37°C for 30 min. At last 50~100*μ*l of DAB and methyl green were added to the mixture. The sections were observed under a fluorescent microscope.

#### 2.4.7. Determination of MAPK-Related Protein Expression by Western Blot Assay

The pulmonary tissues were catalyzed by a proteinase inhibitor, RIPA buffer. Protein concentration was tested by the BCA test kit, SDS added, boiled for 10 min, and then stored at -80°C. The protein was separated by 8%~12% SDS PAGE and then transferred to the PVDF membrane. The non-specific antigen was sealed by 5% of BSA for 1.5 h. Diluted corresponding antibody (1:1000) was then added and the mixture was left overnight at 4°C. The membrane was washed three times of 10 min at room temperature with 1% of TBST. The second antibody (1:2000) was then added and incubated at room temperature for 1h. The membrane was washed three times of 10 min at room temperature with 1% of TBST again. Color-developing reagent was then added and the membrane was exposure to light in dark room.

### 2.5. Statistic Analysis

All data were analyzed by the SPSS 17.0 software package. The metering data were expressed as mean value ± standard error. Between-group difference was analyzed by ANOVA, followed by LSD-t for multiple comparison if significant difference was found.* P* < 0.05 was considered as statistic significance.

## 3. Results

### 3.1. General Information of Mice

0-7 day: The mental state and fur of mice in the normal group did not change markedly, while a majority of mice in any other groups crouched, moved occasionally, and were accompanied by sporadically cough.

8-14 day: No unusual mental state or fur was observed in mice in the normal group. The appetite of mice in the model group declined continuously, accompanied by continuous weariness. Although a part of mice in other groups showed mental weariness, it was improved to a certain degree, their appetite was increased, cough and choke hardly occurred, and furs did not show marked difference.

15-21 day: No unusual mental state or fur was observed in the normal group of rice. A part of mice in the model group still showed mental weariness, but their fur did not show any unusual symptom. The mental state of mice in other groups was improved markedly. No choke or cough occurred and their fur was as usual.

22-28 day: The mental state and fur of mice in the normal group were as usual. A part of mice in the model group still showed mental weariness, but no unusual symptom was observed in their fur. The mental state, fur, and appetite in other groups were as usual as regular.

### 3.2. Body Weight Changes of Mice

**Figure 1:**
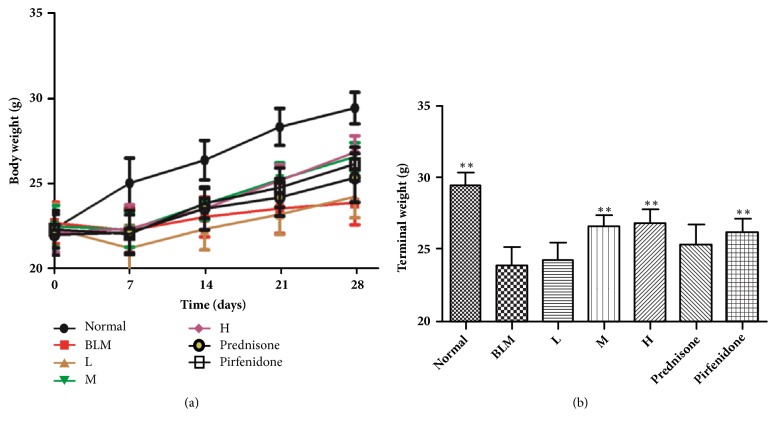
Effects of CAE on mice body weight at different groups. (a) Effects of CAE on mice body weight at different time points. (b) Effects of CAE on terminal weight in BLM-induced mice. Normal: normal group, water; BLM: model group, BLM+water; L: CAE-16 group, BLM+ CAE at a dose of 16mg/kg once per day; M: CAE-32 group, BLM+ CAE at a dose of 32mg/kg once per day; H: CAE-64 group, BLM+ CAE at a dose of 64mg/kg once per day; Prednisone: prednisone group, BLM+ prednisone at a dose of 6mg/kg once per day; Pirfenidone: pirfenidone group, BLM+ prednisone at a dose of 100mg/kg once per day. All data are expressed as mean ±SD (n=6), *∗∗P* < 0.01 versus BLM group.

As shown in Figure 1(a), body weight of mice in any other groups decreased within one week after model construction except for the normal group. But one week after model construction their body weights kept increasing. As shown in Figure 1(b), the final weight of mice in the model mouse group decreased markedly compared to that of the normal group. Although the body weight of mice in the treatment group was increased compared to the model group, statistically significant difference was observed in the high CAE-dosed group, medial CAE-dosed group and Prednisone group.

### 3.3. Pulmonary Alveolitis and Pulmonary Fibrosis Score

**Figure 2:**
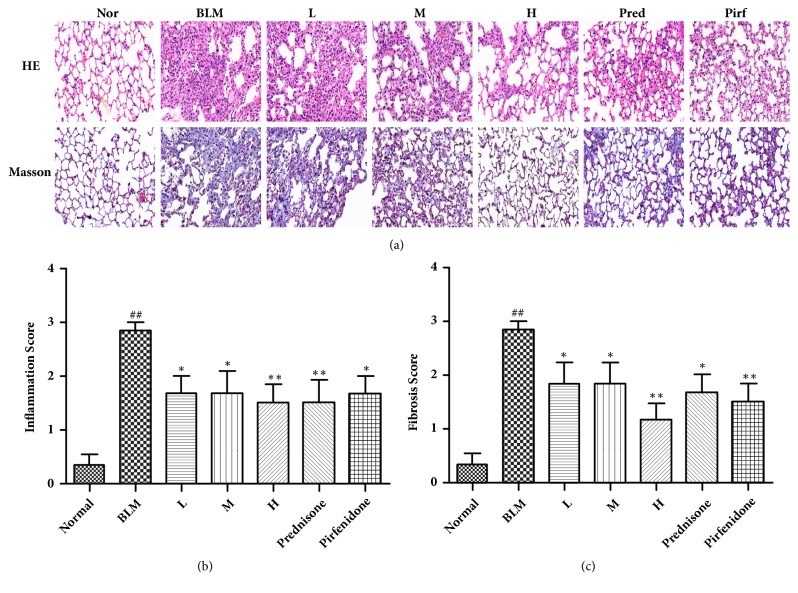
Effects of CAE on histopathologic change of lungs by BLM-induced pulmonary fibrosis mice. (a) Lung sections were stained with HE and Masson, and photographs were taken at magnifications of 200x, respectively. (b) Effects of CAE on the alveolitis score of lung. (c) Effects of CAE on the fibrosis score of lung. Normal: normal group, water; BLM: model group, BLM+water; L: CAE-16 group, BLM+ CAE at a dose of 16mg/kg once per day; M: CAE-32 group, BLM+ CAE at a dose of 32mg/kg once per day; H: CAE-64 group, BLM+ CAE at a dose of 64mg/kg once per day; Prednisone: prednisone group, BLM+ prednisone at a dose of 6mg/kg once per day; Pirfenidone: pirfenidone group, BLM+ prednisone at a dose of 100mg/kg once per day. All data are expressed as mean ±SD (n=6), ^##^P < 0.01 versus Normal group; *∗P* < 0.05 versus BLM group; *∗∗P* < 0.01 versus BLM group.

We calculated the score of pulmonary fibrosis and pulmonary alveolitis based on HE and Masson and then assessed the degree of pulmonary fibrosis. As shown in Figure 2(a) by HE staining, the normal group showed normal pulmonary tissue structure, while the model group showed significantly widened gap between pulmonary alveolus, accompanied by massive inflammatory cellular infiltration, collapsed pulmonary alveolus fusion, and disorganized structure. The gap between pulmonary alveolus of mice in the prednisone group and pirfenidone group was only slightly thickened, accompanied by a handful of inflammatory cellular infiltration. Significant improvement was observed in the high and medial CAE-dosed groups compared to the model group, while no marked improvement was found in the low CAE-dosed group. Meanwhile, as shown in Figure 2(a) by Masson staining, a small quantity of blue staining reaction was observed in the pulmonary tissues of mice in the normal group. A large quantity of blue collagenous fiber was observed in the pulmonary tissues of mice in the model group, and severe collagen deposition occurred. In contrast, only a small quantity of blue collagen was found in the prednisone group and pirfenidone group. Collagen deposition in the high and medial CAE-dosed groups was also significantly improved compared to the low CAE-dosed group.

As shown in Figure 2(b), the score of pulmonary alveolitis and pulmonary fibrosis of mice in the high and medial CAE-dosed group was significantly reduced compared to the model group. The improvement was more significant in high CAE-dosed group. But no marked change was observed in the low CAE-dosed group.

### 3.4. Collagen Content in Pulmonary Tissue

**Figure 3:**
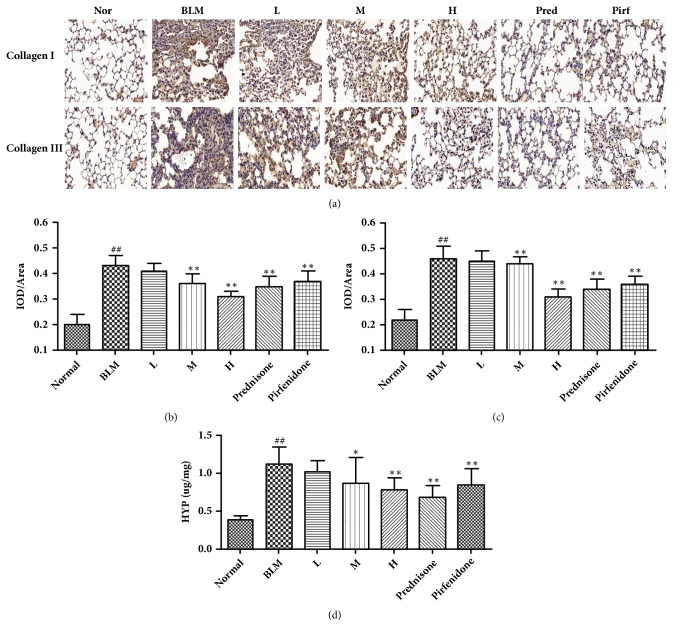
Effects of CAE on collagen content change of BLM-induced lung injury in mice. (a) The expressions of collagen I and collagen III in the lung tissues were measured by immunohistochemical assay (Magnification, 200x). (b) The quantitative result of collagen I. (c) The quantitative result of collagen III. (d) The expressions of HYP in the lung tissues were detected by ELISA method. Normal: normal group, water; BLM: model group, BLM+water; L: CAE-16 group, BLM+ CAE at a dose of 16mg/kg once per day; M: CAE-32 group, BLM+ CAE at a dose of 32mg/kg once per day; H: CAE-64 group, BLM+ CAE at a dose of 64mg/kg once per day; Prednisone: prednisone group, BLM+ prednisone at a dose of 6mg/kg once per day; Pirfenidone: pirfenidone group, BLM+ prednisone at a dose of 100mg/kg once per day. All data are expressed as mean ±SD (n=6), ^##^*P* < 0.01 versus normal group; *∗P* < 0.05 versus BLM group; *∗∗P* < 0.01 versus BLM group.

The basic pathogenic change of pulmonary fibrosis is collagen deposition. The dominant collagens in pulmonary fibrosis are collagen I and collagen III. In addition, HYP is one of the main components of collagens tissue, and its content is an effective indicator to assess collagen deposition in pulmonary tissue [12]. As shown in Figures 3(a), 3(b), and 3(c) by the immunohistochemical assay, the expression of collagen I and collagen III in the mice of the model group was markedly increased compared to the normal group. In contrast, the expression of collagen I was significantly reduced in the high CAE-dosed group, medial CAE-dosed group, prednisone group, and pirfenidone group compared to the model group. Meanwhile, the expression of collagen III was significantly reduced in the high CAE-dosed group, prednisone group, and pirfenidone group compared to the model group. As shown in Figure 3(d) by ELISA method, HYP in the pulmonary tissue of mice in the model group was markedly increased compared to the normal group. However, when mice were treated by prednisone, pirfenidone, and medial and high dosage of CAE, HYP content markedly decreased.

### 3.5. Levels of Inflammatory Factors in Pulmonary Tissue, Serum, and BALF

**Figure 4:**
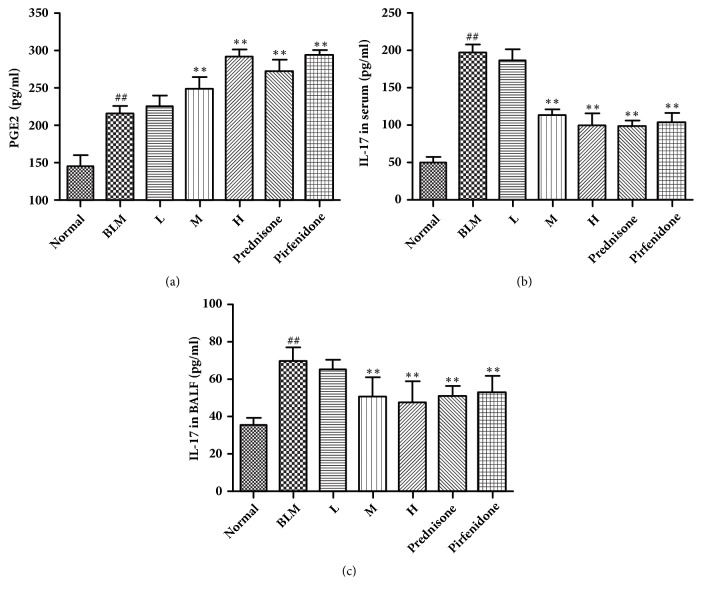
Effects of CAE on the levels of inflammatory factors change of BLM-induced pulmonary fibrosis mice. (a) The content of PGE2 in the lung tissues were determined by ELISA. (b) The content of IL-17 in the serum were measured by ELISA. (c) The contents of IL-17 in the BALF were detected by ELISA. Normal: normal group, water; BLM: model group, BLM+water; L: CAE-16 group, BLM+ CAE at a dose of 16mg/kg once per day; M: CAE-32 group, BLM+ CAE at a dose of 32mg/kg once per day; H: CAE-64 group, BLM+ CAE at a dose of 64mg/kg once per day; Prednisone: prednisone group, BLM+ prednisone at a dose of 6mg/kg once per day; Pirfenidone: pirfenidone group, BLM+ prednisone at a dose of 100mg/kg once per day. All data are expressed as mean ±SD (n=6), ^##^*P* < 0.01 versus normal group; *∗∗P* < 0.01 versus BLM group.

It has been proven that cellular factors play a crucial role in the occurrence and development of pulmonary fibrosis. Cellular factors are regulated reciprocally, constituting a complex cellular factors network and participating into the progression of pulmonary fibrosis [13]. It is conventionally believed that PGE2 has driven the progress of pulmonary fibrosis [14]. However, recent results confirmed that its role in pulmonary fibrosis is protective which inhibits the development of pulmonary fibrosis. We determined PGE2 content in the mice of every group by ELISA method. Our results showed that PGE2 was highly expressed in the mice of model group. Similarly, when mice were treated with different concentrations of CAE, the expression of PGE2 increased with increasing CAE concentrations. Particularly, PGE2 contents in the pulmonary tissues of the medial and high CAE-dosed mice group, prednisone group, and pirfenidone group were significantly increased compared to the model group (Figure 4(a)).

It has been confirmed that IL-17 accelerates the progression of pulmonary fibrosis in various pathways [15]. To confirm the inhibitory role of CAE on IL-17, we adopted the ELISA method to test the variations of IL-17 levels in serum and BALF. Our results showed that IL-17 levels in serum and BALF of the model group increased significantly compared to the control group. In contrast, IL-17 levels in the high and medial CAE-dosed groups, prednisone group, and pirfenidone group decreased significantly compared to the model group (Figures 4(b) and 4(c)).

### 3.6. Expression of Related Marker Proteins in Pulmonary Tissue

**Figure 5:**
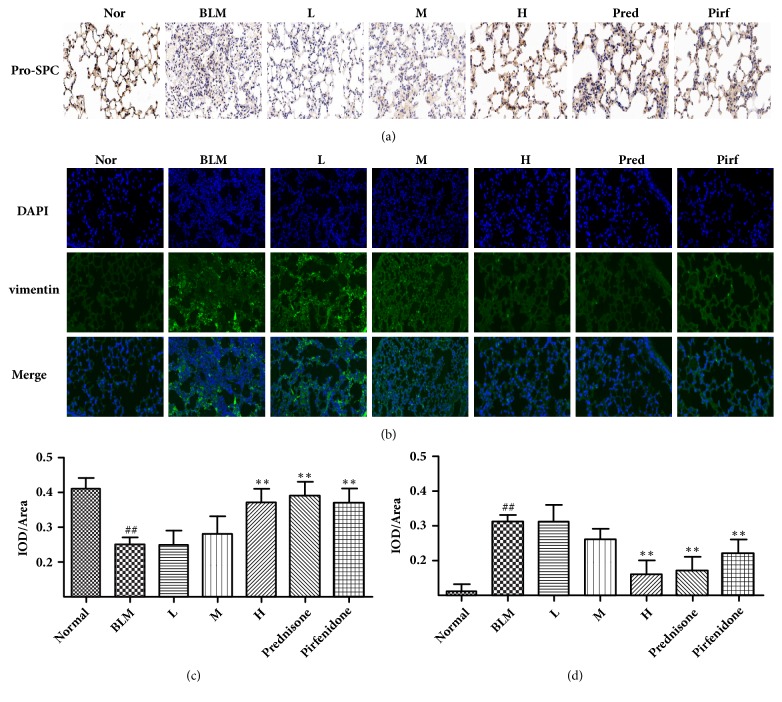
Effects of CAE on the expression of related marker proteins in pulmonary tissue of BLM-induced pulmonary fibrosis mice. (a) The expressions of Pro-SPC in the lung tissues were measured by immunohistochemical assay (Magnification, 200x). (b) The expressions of vimentin in the lung tissues were determined by immune-fluorescent methods (Magnification, 200x). (c) The quantitative result of Pro-SPC. (d) The quantitative result of vimentin. Normal: normal group, water; BLM: model group, BLM+water; L: CAE-16 group, BLM+ CAE at a dose of 16mg/kg once per day; M: CAE-32 group, BLM+ CAE at a dose of 32mg/kg once per day; H: CAE-64 group, BLM+ CAE at a dose of 64mg/kg once per day; Prednisone: prednisone group, BLM+ prednisone at a dose of 6mg/kg once per day; Pirfenidone: pirfenidone group, BLM+ prednisone at a dose of 100mg/kg once per day. All data are expressed as mean ±SD (n=6), ^##^*P* < 0.01 versus normal group; *∗∗P* < 0.01 versus BLM group.

The basic pathogenic changes of pulmonary fibrosis are the reduced number of alveolar epithelial cells and massive proliferation of lung fibroblast. Because both Pro-SPC and vimentin can act as the alveolar epithelial cells and pulmonary fibroblasts specific proteins [16, 17], we therefore could evaluate the degree of pulmonary fibrosis by determining their amount of expression via immune-histochemical and immune-fluorescent methods. As shown in Figures 5(a) and 5(c), the Pro-SPC expression in model mice group was significantly reduced compared to the normal mice group. In contrast, although it was markedly increased in the high CAE-dosed group, prednisone group, and pirfenidone group compared to the model group, the proportion was still higher than the normal group. As shown in Figures 5(b) and 5(d), green fluorescence represents vimentin expression, while the blue fluorescence represents DAPI-stained nuclei. According to the results of immunofluorescence, the expression of vimentin in the model mouse group increased significantly compared to the normal group. In contrast, it was reduced to a certain degree after drug administration. In particular, it was reduced clearly in the medial and high CAE-dosed group, prednisone group and pirfenidone group. The results above suggested that, after successful model construction, the quantity of alveolar epithelial cells decreased gradually, while that of pulmonary fibroblasts increased massively. The intervention of CAE however has inhibited alveolar epithelial cells damage and pulmonary fibroblasts proliferation to a certain degree.

### 3.7. Cell Apoptosis in Pulmonary Tissue

**Figure 6:**
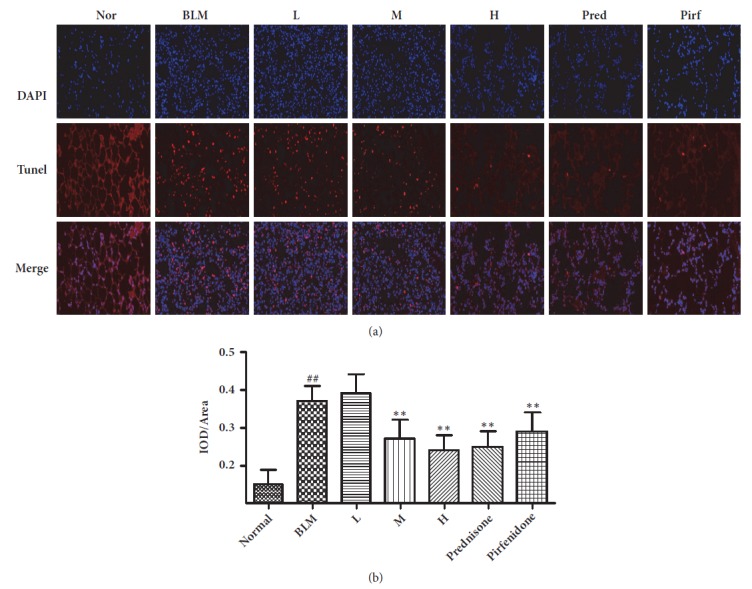
Effects of CAE on the cell apoptosis in pulmonary tissue of BLM-induced pulmonary fibrosis mice. (a) The apoptotic cells in pulmonary tissue were stained by the Tunel test kit and observed with a fluorescence microscope. (Magnification, 200x). (b) The quantitative result of TUNEL staining. Normal: normal group, water; BLM: model group, BLM+water; L: CAE-16 group, BLM+ CAE at a dose of 16mg/kg once per day; M: CAE-32 group, BLM+ CAE at a dose of 32mg/kg once per day; H: CAE-64 group, BLM+ CAE at a dose of 64mg/kg once per day; Prednisone: prednisone group, BLM+ prednisone at a dose of 6mg/kg once per day; Pirfenidone: pirfenidone group, BLM+ prednisone at a dose of 100mg/kg once per day. All data are expressed as mean ±SD (n=6), ^##^*P* < 0.01 versus normal group; *∗∗P* < 0.01 versus BLM group.

It has been confirmed that with the pathogenic development of pulmonary fibrosis, the number of apoptotic cells increased gradually, which to a certain degree reflects the pathogenic degree of pulmonary fibrosis [18]. We adopted Tunel method, a frequently used method for tissue apoptosis test, to investigate the role of CAE in the inhibition of cell apoptosis in pulmonary tissues. As shown in Figure 6(a), red fluorescence represents apoptotic cells and blue fluorescence indicates cellular nuclei. Because cell apoptosis occurs frequently in cellular nuclei, the superposition of red and blue fluorescence therefore represents apoptotic cells. The results of Tunel assay showed that the number of apoptotic cells in the model group was increased significantly compared to the normal group. In contrast, it was significantly reduced in the high CAE-dosed group, prednisone group, and pirfenidone group compared to the model group (Figure 6(b)).

### 3.8. Expression of p-p38 and NF-*κ*B in Pulmonary Tissue

**Figure 7:**
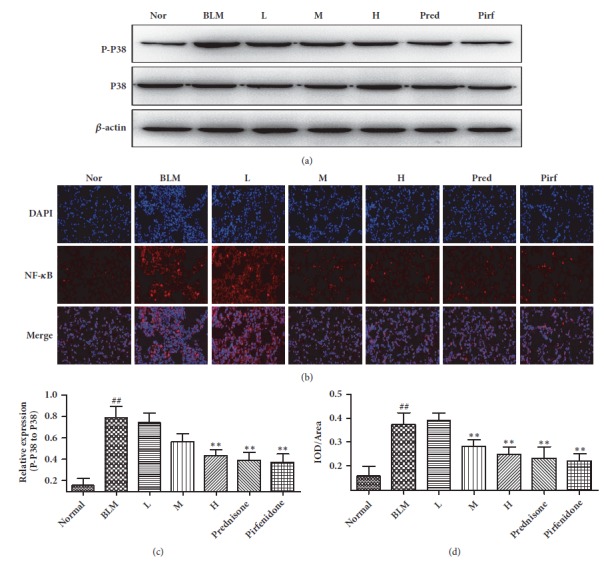
Effects of CAE on the expression of p-p38 and NF-*κ*B in pulmonary tissue of BLM-induced pulmonary fibrosis mice. (a) The expressions of p-p38 in the lung tissues were recorded by Western Blot. (b) The expressions of NF-*κ*B in the lung tissues were tested by immune-fluorescent methods (Magnification, 200x). (c) The quantitative result of p-p38. All data are expressed as mean ±SD (n=3), ^##^*P* < 0.01 versus normal group; *∗∗P* < 0.01 versus BLM group. (d) The quantitative result of NF-*κ*B. All data are expressed as mean ±SD (n=6), ^##^*P* < 0.01 versus normal group; *∗∗P* < 0.01 versus BLM group. Normal: normal group, water; BLM: model group, BLM+water; L: CAE-16 group, BLM+ CAE at a dose of 16mg/kg once per day; M: CAE-32 group, BLM+ CAE at a dose of 32mg/kg once per day; H: CAE-64 group, BLM+ CAE at a dose of 64mg/kg once per day; Prednisone: prednisone group, BLM+ prednisone at a dose of 6mg/kg once per day; Pirfenidone: pirfenidone group, BLM+ prednisone at a dose of 100mg/kg once per day.

The role of cell apoptosis in pulmonary fibrosis involves with several signaling pathways. The MAPK/NF-*κ*B signaling pathway probably plays a crucial role [19]. We firstly determined the expression of MAPK-related signaling pathways, P-ERK, P-JUK, and P-P38 by the Western Blot method [20]. The results showed that only P-P38 have played a role. The expression level of P-P38 in the pulmonary tissues of model mouse group was increased significantly compared to the control group. In contrast, after drug administration, the expression of P-P38 was clearly reduced. Of which, the changes in high CAE-dosed group, prednisone group and pirfenidone group were more than other group (Figures 7(a) and 7(c)).

In addition, we determined the amount and site of NF-*κ*B expression by immunofluorescence method. As shown in the Figures 7(b) and 7(d), red fluorescence presents NF-*κ*B expression, blue fluorescence shows cellular nuclei staining and overlapping part shows the entrance of NF-*κ*B into cellular nuclei. Results of immunofluoresence histochemical assay showed that the expression level of NF-*κ*B in the pulmonary tissues of the model mouse group was significantly increased compared to the control group, and that the number of NF-*κ*B entrance into the cellular nuclei was also markedly increased. In contrast, after drug administration the expression level of NF-*κ*B and the number of NF-*κ*B entered into cellular nuclei were both reduced. In particular, the changes of the high CAE-dosed group and prednisone group were more significant.

## 4. Discussion

Pulmonary fibrosis is a progressive lethal disease with increasing annual incidence, and no effective drug has been developed for pulmonary fibrosis treatment as yet [21]. Therefore, it is of important clinical value to search for drugs with high safety and low side effects. Our previous research showed that CAE can prevent and cure BLM-induced pulmonary fibrosis rats and inhibit the pulmonary fibroblast of human embryo* in vitro*. In this paper, we constructed BLM-induced pulmonary fibrosis model mice, tested the curative effect of CAE, and further confirmed its functioning mechanism.

Pulmonary fibrosis model can be constructed by a variety of methods, including Paraquat- and Amiodarone-induced ones. But disposable BLM instillation is the most frequently used method at present, and prednisone is the mostly selected drug for positive control studies [22]. Pirfenidone is one of the poorly recommended drugs in the IPF handbook. Its curative effect and safety are not clear, so we also supplemented it to the positive control drug to test its protecting role in the BLM-induced pulmonary fibrosis mice. To test if pulmonary fibrosis mouse model has been constructed successfully, we conducted pathogenic test of the embedded pulmonary tissues by HE and Masson and further analyzed them by the score of pulmonary alveolitis and pulmonary fibrosis. Results showed that the score of pulmonary alveolitis and pulmonary fibrosis in the model mouse group was significantly increased compared to the normal group. In contrast, it was markedly reduced in the medial and high CAE-dosed group, prednisone group, and pirfenidone group, suggesting a successful construction of pulmonary fibrosis mouse model. Meanwhile, CAE, prednisone and pirfenidone markedly ameliorated BLM-induced pulmonary fibrosis mouse model. In addition, successful model construction and amelioration in pulmonary fibrosis mouse model can also be reflected indirectly by mice body weight variation.

The basic pathogenic trait of pulmonary interstitial fibrosis is the mass deposition of extracellular matrix that may affect the structure of normal pulmonary tissues. Therefore collagen content reflects the severity of pulmonary fibrosis to a certain degree [23]. It has been found that collagen is one of the components of extracellular matrix (ECM). There are four kinds of collagens in pulmonary tissues, and of which collage I and collagen III are the dominant collagens. In addition, collagen contains abundant HYP. Therefore, expression of collagen I and collagen III and HYP content can reflect the situation of collagen deposition. Our results showed that high CAE dosage could inhibit collagen I, collagen III, and HYP, which also suggests the protecting role of CAE in BLM-induced pulmonary fibrosis mice in another point of view.

Although the pathogenic mechanism of pulmonary fibrosis remains unclear, available results have proven the participation of various cellular factors into pulmonary fibrosis. Traditional viewpoint believes that PGE2 is a molecule that promotes inflammatory response and accelerates the development of pulmonary fibrosis. Recent findings showed that PGE2 functions to fight against inflammation instead of promoting inflammation. PGE2 plays its anti-inflammatory effect and protecting role in the development of pulmonary fibrosis through proliferation of CD and T cells, reduced releases of inflammatory factors, inhibited activation and migration of macrophagocytes [24–26]. Our study found that PGE2 was highly expressed in the model mice group. When mice were dosed by different concentrations of CAE, PGE2 concentration was increased in a concentration-dependent manner. PGE2 contents in the pulmonary tissue of mice in the high CAE-dosed group, prednisone group, and pirfenidone group were also increased significantly. In the present study, PGE2 level in the model group was higher than the normal group. It was reported that in the BALF of IPF patients PGE2 was significantly reduced compared to the control [27]. This is probably attributed to the animal model itself. Although BLM-induced pulmonary fibrosis model mice were used in pulmonary fibrosis studies, it is more close to an acute pulmonary damage model. The inflammatory response occurs throughout the process. Therefore, although lung tissue was sampled 28 days after model construction, inflammatory compensatory response still exists.

IL-17 is a secretory protein. It has been confirmed that IL-17 probably has participated into the developmental progress of pulmonary fibrosis [28]. Therefore, IL-17 can reflect the pathogenic degree of pulmonary fibrosis to a certain degree. IL-17 not only regulates the oxidative induction, apoptosis, autophagy, and other biological effects by mediating the MAPK, GSK3*β*, NF-*κ*B, and other related signaling pathways but also plays role synergistically with other related cellular factors, and accelerate the development of pulmonary fibrosis [29]. Wilson et al. [15] found that IL-17 can function in combination with TGF-*β*1 to promote the synthesis and secretion of collagen and accelerate the occurrence and development of pulmonary fibrosis by constructing different* in vivo* pulmonary fibrosis model. The present study found that CAE can reduce the content of IL-17 in pulmonary fibrosis mice serum and BALF, suggesting that CAE probably can inhibit the inflammatory response and delay the development of pulmonary fibrosis.

The basic pathogenic change of pulmonary fibrosis is characterized by reduced quantity of alveolar epithelial cells and massive proliferation of pulmonary fibroblasts. SPC is a specific protein of alveolar epithelial cells. Because its activity is unstable, we detected Pro-SPC by the immunohistochemical method instead of SPC. Vimentin is normally expressed in mesenchymal tissue, not in epithelial cells. Therefore, it can be a marker protein of pulmonary fibroblasts. We can determine the expression of vimentin in pulmonary tissue by the immunofluorescent method. We found in the present study that the proportion of cells with positive Pro-SPC expression was markedly reduced in the model mice group than the normal group. In contrast, although the proportion in the high CAE-dosed group, prednisone group, and pirfenidone group was markedly increased compared to the model group, it was still lower than the normal group. Meanwhile, the results of immunofluorescence study showed that the proportion of cells with positive vimentin expression was markedly increased in the model group than the normal group. In contrast, the proportion was reduced to a certain degree when mice were dosed by different drugs. In particular, the proportion was mostly reduced in the high CAE-dosed group, prednisone group and pirfenidone group. The results above suggested that, after successful model construction, the quantity of alveolar epithelial cells decreased gradually while that of pulmonary fibroblasts increased massively. In contrast, when mice were treated with CAE, prednisone, and pirfenidone, the damage of alveolar epithelial cells and proliferation of pulmonary fibroblasts was inhibited to a certain degree.

Cell apoptosis is one of ways of programmed cell death, which is mainly characterized by cell shrinkage, nuclei karyopyknosis, formation of apoptotic body, and DNA fragmentation [30]. It has been showed that the role of pulmonary fibrosis in cell apoptosis involves two ways. One is the excessive apoptosis of alveolar epithelial cells. Alveolar epithelial cells damage is probably the early event of diseases. The damaged alveolar epithelial cells can secrete a number of cellular factors including TGF-*β*1, which accelerates the progress of pulmonary fibrosis [31]. Another is the insufficient apoptosis of pulmonary fibroblasts. Pulmonary fibroblasts have distinct apoptosis resistance, and insufficient apoptosis of pulmonary fibroblasts can promote its massive proliferation and conversion to myofibroblasts, secrete a plenty of collagen, and accelerate the occurrence and development of pulmonary fibrosis [32]. Results of our study showed that the number of apoptotic cells in the model mice group was markedly increased compared to the normal mice group. In contrast, it was significantly reduced in the pulmonary tissues of the high CAE-dosed mice group, prednisone group, and pirfenidone group compared to the model mice group. This suggested that CAE can function to resist pulmonary fibrosis by inhibiting the cell apoptosis of pulmonary fibrosis mice.

The role of cell apoptosis in pulmonary fibrosis involves with several signaling pathways. It has been shown that the MAPK/NF-*κ*B signaling pathway probably plays a crucial function in pulmonary fibrosis. The MAPK signaling pathway consists of a category of important growth regulation proteins that play a pivotal role in the transfer of cytoplasmic and cell nuclear signals and regulate physiological process related to cellular proliferation, differentiation and immune response [33]. The MAPK family includes mainly three members, p38, JNK, and ERK. NF-*κ*B is an important transcriptional regulator that controls inflammatory reaction. It usually forms a complex with the inhibitory protein I*κ*B and is present in cytoplasm. Once stimulated by various stimuli, the extracellular signals were transmitted into cells, which probably activates and phosphorates I*κ*B and further promotes the separation of the NF-*κ*B/I*κ*B complex [34]. The free NF-*κ*B can enter the cell nucleus rapidly and then binds specifically to related domain of the promoter of target gene, thereby activating the target gene and promoting the transcription of related proteins. It has been confirmed that with the progression of pulmonary fibrosis one or both pathways of the MAPK signaling pathways were activated, which further promoted NF-*κ*B to enter the cell nucleus and initiated the transcription of cell apoptosis-related proteins [20]. We found in the present study that the expression levels of NF-*κ*B and p38 and the number of NF-*κ*B entered into cell nucleus in the pulmonary tissues of the model mice group were both significantly increased compared to the control group and of which the changes in the high CAE-dosed group, prednisone group, and pirfenidone group were most significant. This suggests that role of CAE in BLM-induced pulmonary fibrosis mice is probably related to the inhibition of the NF-*κ*B/p38 signaling pathway.

## 5. Conclusions

Taken together, CAE could effectively delay the advancement of BLM-induced pulmonary fibrosis in mice, which is probably attributed to inhibition of collagen secretion, prevention of inflammatory response, protection of pulmonary epithelial cells, and inhibited proliferation of pulmonary fibroblasts. A possible mechanism underlying is the inhibition of p38/NF-*κ*B signaling pathway. However, indepth investigation is necessary to conclude a more specific functioning mechanism.

## Data Availability

The data used to support the findings of this study are available from the corresponding author upon request.
